# Diagnostic performance of the Qiaprep amp Viral RNA UM kit for the detection of COVID-19 compared to RT-PCR

**DOI:** 10.3389/fmed.2022.976090

**Published:** 2022-10-06

**Authors:** Eduardo Becerril Vargas, Gabriel Cojuc-Konigsberg, Alan Braverman-Poyastro, Erick Armendáriz Mendoza, Mario Alberto Mujica Sánchez, María Del Carmen García Colín, Hansel Hugo Chávez Morales, José Nicolás Aguirre Pineda, Luis Carlos Ibarra Cobas

**Affiliations:** Clinical Microbiology Laboratory, National Institute of Respiratory Diseases, Mexico City, Mexico

**Keywords:** PCR real time (qPCR), cycle threshold (Ct) value, SARS–CoV−2, amplification, Qiaprep

## Abstract

**Background:**

RT-PCR is the currently recommended laboratory method for diagnosing acute SARS-CoV-2 infection. Nevertheless, to carry out this assay, numerous manual steps are necessary, but they are long lasting and error-prone. A new sample preparation solution was launched, the Qiaprep & amp Viral RNA UM kit, that combines a short, liquid-based sample preparation with one-step RT-PCR amplification and detection of SARS-CoV-2. Such alternative allows reducing the handling of samples and obtaining a result in a shorter period of time. The objective of the study was to compare the performance of the kit with RT-PCR.

**Methods:**

A prospective trial was carried out in the clinical microbiology laboratory of a tertiary care hospital. The pharyngeal and nasopharyngeal swabs included in the study were taken from patients who underwent medical consultation because compatible COVID-19 symptoms. Samples were processed simultaneously for the reference RT-PCR and by the QIA P&A kit.

**Results:**

190 samples were included in the clinical trial. The reference RT-PCR method indicated that 125 (66%) samples, out of the 190, were positive. The QIA P&A kit showed 112 positive samples for SARS-CoV-2. The QIA P&A kit has a sensitivity of 86% to detect SARS-CoV-2 and a 100% specificity, the positive predictive value was of 96%, the negative predictive value 78%, and the obtained Kappa value was 0,76. QIA P&A kit showed a lower mean cycle threshold compared with the diagnostic standard, with a statistically significant difference (*p* < 0.05).

**Conclusion:**

The QIA P&A kit has an acceptable, yet not optimal performance for sample preparation and amplification of SARS-CoV-2 and further studying is required for it to be validated as a cost-effective, rapid diagnostic method for detecting infections.

## Introduction

Coronavirus is an enveloped RNA virus that causes severe acute respiratory syndrome (SARS-CoV-2). It belongs to the *Coronaviridae* family, the etiological agent of coronavirus disease (COVID-19) (1). In the first sequenced genome of the virus, the 16 main open reading frames (ORF) corresponding to ORF1a and ORF1b were identified, encoding 16 non-structural proteins (nsp) and 4 main structural proteins: surface (S), membrane (M), envelope (E) and nucleocapsid (N). Early diagnosis and isolation of suspected patients play a vital role in controlling the pandemic, which has prompted a large number of diagnostic test manufacturers during the initial phase to address the design, development, validation, verification and implementation of tests for the identification of this virus. Currently, there are many commercial SARS-CoV-2 detection kits, which have been authorized for use by the FDA and Mexican authorities. These tests can identify viral genetic regions using specific nucleic acid amplification techniques such as real-time reverse transcription polymerase chain reaction (RT-PCR), which is currently considered the gold standard for diagnosis confirmation (2, 3).

Nucleic acid amplification tests detect unique viral RNA sequences within genes that code for N, E, S proteins or RNA polymerase (4). Synthetically produced complementary RNA sequences act as primer sequences when coupled to viral RNA, after which they are amplified by means of enzymatic reactions that involve high-efficiency RNA polymerases resistant to infection. temperature and cofactors that are mixed in reactions with thermocycling. Finally, quantitative reading of RNA amplification is performed. RT-PCR was the first diagnostic method applied for the diagnosis of COVID-19 (5, 6). The time to obtain a result from the time a sample is obtained for diagnosis by RT-PCR depends on several factors including storage time and sample transport to a clinical diagnostic laboratory.

RNA extraction yields samples of optimal quality for analysis. Nonetheless, this step could be omitted to shorten times, although the optimal diagnostic performance of the test would appear to be sacrificed. Rapid methods are currently recommended in patients with a high pretest probability (i.e., symptomatic individuals, patients with risk factors and areas with significant active transmission of the disease) in areas with a high demand for diagnostic tests, with the objective of optimizing diagnostic processes (7).

The RNA extraction process impacts the diagnostic performance of RT-PCR for SARS-CoV-2 (8). Existing evidence suggests that the omission of RNA extraction increases the value of the cycle threshold (Ct) to reach the detection threshold of RT-PCR compared to processing by RNA extraction. The samples processed with RNA extraction had an overall sensitivity > 90%, omitting extraction resulted in a sensitivity of 51–62% (9).

The increase in demand for diagnostic tests has led to a delay in diagnosis. In most laboratories and diagnostic centers RT-PCR tests require time and specialized personnel to carry out this type of tests. Numerous steps of manual work are necessary taking approximately 6 to 8 h to obtain a result. Processing oropharyngeal/nasopharyngeal swab samples with the Qiaprep & amp Viral RNA UM kit (QIA P&A) takes less than an hour, includes an optional heat treatment step, and only three pipetting steps performed directly into the PCR reaction vessel. This streamlined procedure reduces the number of handling steps and the use of plastic and can be fully automated with liquid handlers (10).

The aim of this study was to compare the performance of the QIA P&A with the current diagnosis standard for SARS-CoV-2 at a high-volume reference center for respiratory and infectious diseases in Mexico City, Mexico.

## Materials and methods

### Study design and participants

A prospective clinical trial was carried out in the clinical microbiology laboratory of a national reference medical center. Samples from patients in whom COVID 19 was clinically suspected and for whom RT-PCR was requested for the detection of SARS-CoV-2 were included. Samples reported as non-compliant product based on clinical microbiology laboratory guidelines were excluded. The samples were taken by a chemist with appropriate training. Nasopharyngeal and pharyngeal exudates were obtained and both swab samples were set in a plastic tube with 5 mL of Universal Transport Medium. All samples were processed for RT-PCR using the reference method established in the microbiology laboratory and with the QIA P&A kit. Sample processing was performed by both methods after collection.

### RT-PCR technique

The first step for conventional RT-PCR technique is RNA extraction. Briefly, 200 μl of each oropharyngeal/nasopharyngeal exudate sample were added to an individual well in the extraction plate of the kit BIONEER ExipPrep 96 Viral DNA/RNA (Ref. K-4614) and a total nucleic acid extract (DNA and RNA) was obtained automatically in the BIONEER Exiprep equipment according to the manufacturer's specifications. Retrotranscription and PCR were performed with the GeneFinderTM COVID-19 Plus RealAmp RT-PCR kit (Ref. IFMR-45), which detects the presence of the RdRP, N and E genes of SARS-COV2 virus. This process was compliant the manufacturer's specifications. The reaction mix was made by combining 10 μL of the master mix and 5 μL of the probe mix. In the end, 5 μL of the nucleic acid extract was added for each sample, resulting in a final volume of 20 μL. RT-PCR was performed in a Quant Studio 5 thermocycler (Applied Biosystems) under the following amplification conditions: 50°C/20 min, 95°C/5 min, followed by 45 cycles of 95°C/15 sec and 58°C/60 sec.

### QIA P&A kit

For processing with the QIA P&A kit RNA, amplification was performed according to the manufacturer's specifications. An aliquot of 8 μL from each of the oropharyngeal/nasopharyngeal swab samples contained in universal transport medium was mixed with 2 μL of Viral RNA UM Prep Buffer and then it was added to an optimized sample preparation buffer, as described in Table 1. The tubes containing the final reaction volume (20 μL) were set in a real-time cycler and amplification was performed as described in Table 2.

**Table 1:** Components of the reaction mix for the Qiaprep amp Viral RNA UM kit.

**Component**	**Volume**
Viral RNA master mix, 4x	5 μL
20x primer–probe mix	1 μL
RNA IC template + assay, 10x	2 μL
Human sampling IC assay, 20x	1 μL
ROX reference dye	1 μL
RNase-free water	Fill up to 10 μL
Prepared sample	10 μL
Total reaction volume	20 μL

**Table 2:** Cycling conditions.

**Step**	**Time**	**Temperature**
Reverse Transcription (RT)	10 min	50 °C
PCR initial heat activation	2 min	95 °C
2-step cycling (40 cycles)
Denaturation	5 s	95 °C
Combined annealing/extension	30 s	58 °C

### Statistical analysis

The collection of results obtained from the processing of the samples by both methods was carried out. Statistical analysis was performed using the statistical package, SPSS 27. Sensitivity and specificity were calculated.

### Ethical considerations

This study follows the ethical guidelines established for the use of patient information and has been approved by INER's ethical committee.

## Results

A total of 190 patient samples were included. The male/female ratio was 1:1.2. All samples with Ct below 40 were reported as positive for the purposes of this study.

### Primary outcome

Of the 190 samples analyzed 125 samples (66%) were reported as positive by RT-PCR and the rest were negative for SARS-CoV-2 (34%). Using the QIA P&A kit, 112 samples were positive for SARS-CoV-2. For the detection of SARS-CoV-2, the sensitivity of the QIA P&A kit was 86%, specificity 94%, with a positive predictive value of 96%, a negative predictive value of 78% and a kappa (k) index of 0.76 (Table 3).

**Table 3:** Comparison between RT-PCR performance (reference method) and QIA P&A.

**QIA P&A**	**RT-PCR (reference method)**		**Sensitivity** **(%)**	**Specificity (%)**	**PPV (%)**	**NPV (%)**	**LR (+)**	**LR (-)**
	**Positive**	**Negative**						
Positive	108	4	86	94	96	78	14.3	0.15
Negative	17	61						

NPV, negative predictive value; PPV, positive predictive value; LR(+), Likelihood Ratio for a positive test; LR(-), Likelihood Ratio for a negative test.

### Cycle thresholds

**Figure 1:**
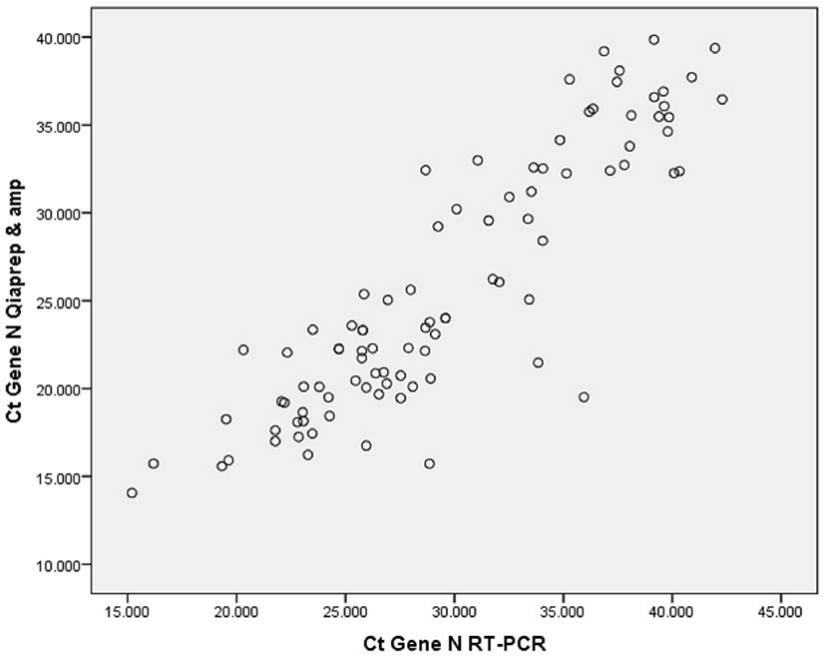
Comparison between real time PCR with QIA P&A and manual PCR assay. The distribution of the Ct gene sample N (confirmatory test) shows a correlation coefficient (r) of 0.45.

The QIA P&A kit showed a lower Ct mean, compared to the manual method, with a statistically significant difference (*p* = 0.001) (Table 4). There was an excellent correlation using the tests, reporting a correlation r = 0.45 (Figure 1). Median CTs of the N gene of samples with positive and negative results obtained with the QIA P&A kit are shown in Table 5. The mean Ct values were lower for the samples that were QIA P&A positive compared to the mean Ct values for the samples that were negative. Median Ct for positive tests was 29.56 (±6.48) compared to 30.82 (±6.98) with no statistically significant difference (*p* = 0.46). The median of the CT for the E and RdRP genes detected by the reference method used in the laboratory were 26.1 ± 6.71 and 30.24 ± 6.72, respectively.

**Table 4:** Median CTs of Gene N by the reference method and QIA P&A.

	**RT–PCR(reference method)**	**QIA P&A**	**p-value**
	Ct ± SD	Ct ± SD	
Gene N	25,86 ± 6,97	25,46 ± 12,2	0,001

Ct, cycle threshold, SD, standard deviation.

**Table 5:** Median CTs of the N gene of samples with positive and negative results obtained with the QIA P&A kit.

	**Ct median–positive samples**	**Ct median–negative samples**	**p-value**
	Ct ± SD	Ct ± SD	
**QIA P&A**	29,56 ± 6,48	30,82 ± 6,98	0,46

CT, cycle threshold; SD, standard deviation.

## Discussion

The timely detection of COVID-19 is essential to reduce the chain of transmission and to optimize hospital processes. An important consequence of the worldwide spread of the virus is shortage of material for molecular diagnostics by suppliers. For this reason, many laboratories implemented new alternative platforms to facilitate timely diagnoses.

The design of this study was planned to establish the diagnostic performance of the QIA P&A test in oropharyngeal/nasopharyngeal samples for the detection of COVID-19 compared to RT-PCR. The observed sensitivity rate of 86% in this study was slightly lower compared to those reported in the existing literature. The most significant was the 87.5–100% reported by Claas ECJ et al. in four hospital laboratories in the Netherlands (11).

Some essential differences observed between the two assays were the use of a smaller volume of sample and reaction mixture with the QIA P&A kit, which does not compromise the quality of the results (11). The strategy allows reduction of the number of samples and other reagents without affecting performance. One of the important advantages of the evaluated method is a possible decrease in errors by the test analysts, since it implies less contact and handling of the samples. The average Ct was lower with the QIA P&A kit, which could indicate a better performance of the evaluated kit, and account for the elimination of numerous steps for RNA extraction and purification. Other authors have observed these results when using automated platforms, and omitting the purification of the samples' genetic material (12).

The limitation of this test is its limited capacity, only detect specific regions of the N gene. In the thirteen samples classified as false negatives by the QIA P&A kit, the N gene was not detected by the standard method. When these samples were subjected to RT-PCR, the E and RDRP genes were detected.

However, with the results obtained in the analysis, negative probability ratio, positive probability ratio and the Kappa index, it can be considered that the QIA P&A kit can optimize the workflow and significantly reduce the time to obtain results. The use of this kit made it possible to considerably increase the number of tests processed per day in the microbiology laboratory of our center, from processing of 1,440 samples to 2,304 samples in 24 h. Nucleic extraction is a crucial part of molecular diagnostic procedures for DNA and RNA purification, this can take anywhere from 30 min to a couple of hours. With the QIA P&A kit this step is skipped, cutting the total time to < 60 min.

This innovative diagnostic test could provide timely diagnoses with acceptable, although not optimal accuracy. It is imperative that techniques are standardized in order to reach higher sensitivities. These tests could optimize massive epidemiological studies and facilitate therapeutic decision making, particularly when highly-transmissible variants are rapidly emerging. Despite the efforts to develop more efficient and effective diagnostic methods for SARS-CoV-2 detection, RT-PCR remains the gold-standard. Further studies are required to assess multi-centric performance, testing method differences, cost-effectiveness analyses and implementation protocols.

## Data availability statement

The original contributions presented in the study are included in the article/supplementary material, further inquiries can be directed to the corresponding author.

## Author contributions

All authors listed have made a substantial, direct, and intellectual contribution to the work and approved it for publication.

## Conflict of interest

The authors declare that the research was conducted in the absence of any commercial or financial relationships that could be construed as a potential conflict of interest.

## Publisher's note

All claims expressed in this article are solely those of the authors and do not necessarily represent those of their affiliated organizations, or those of the publisher, the editors and the reviewers. Any product that may be evaluated in this article, or claim that may be made by its manufacturer, is not guaranteed or endorsed by the publisher.
